# A Charming Medical Personality: the Late Dr. Lytton Maitland

**Published:** 1916-05-13

**Authors:** 


### A CHARMING MEDICAL PERSONALITY.

### The Late Dr. Lytton Maitland.

We are very sorry to record this week the death of Dr. Lytton Maitland, for he was not only a man of professional distinction and attainments, but also a valued contributor to The Hospital. Dr. Maitland, who was an M.D. of London University, took the M.B., B.S. so recently as 1904, and has thus died in the very prime of life. A Charing Cross Hospital man, he held at one time the post of medical registrar to that hospital, and began his connection with medical journalism by editing his hospital's *Gazette*. The old files were treasured by him to the last, and he had some reason to believe that under his editorship the *Gazette* maintained an unusually high level. The son of a medical man, young Dr. Maitland engaged for a time in private practice; but his virtues told against his speedy success. A very modest, quiet, unassuming, and gentle personality like his is not the sort which finds a zest in competitive struggles; and his sense of science, which made his judgments so carefully considered, probably was less apt to attract the average patient than the more assuming dogmatism of many men who were quite destitute of his professional attainments. The simplest proof of his real interest in science can be found in the fact that he taught himself to read and speak German; and his sense of economy being greater than his resources he found no difficulty in spending most of his holidays abroad. He spoke French with ease and fluency, and professional and general literature in French and German were generally to be found in his pocket and always on his table at home. This high standard of general and professional culture, with his natural bent towards the science rather than the art of medicine, led him eventually to seek advancement through the avenue of public health work.

He began to carry out his scheme with characteristic thoroughness. He took the D.P.H. of Cambridge, and after some experience of fever work at, we believe, the Western Hospital, he became, first, assistant and then medical superintendent at the London Fever Hospital. This appointment tested his administrative powers, and proved him, to the delight of his friends, to possess a combination of tact with firmness which had scarcely been expected of so gentle and sympathetic a nature.

After this period of fever work and institutional administration he determined to add to his qualifications for a medical officership of health, practical knowledge of tuberculosis both in its dispensary and sanatorium branches. In pursuance of this plan, therefore, he became medical officer to the Shoreditch Tuberculosis Dispensary connected with the Royal Hospital for Diseases of the Chest. Here he gained much of the clinical experience he wanted and also valuable knowledge of the administrative side of dispensary work. He knew that a medical officer of health must have this if he is to make the most of the dispensary system under him. The subject of rural dispensaries also interested him, and he went into every detail of the cost of construction, the practicability of the travelling and temporary varieties, and the real value, in face of country prejudices, of the different kinds of equipment which before the war were exercising many minds. Finally he acquired sanatorium experience as one of the residents at Winchmore Hill.

This brief outline of Dr. Lytton Maitland's career indicates, indeed, his perseverance, study, and genuine scientific interest in his profession—an interest, be it remembered, not so common as it ought to be. But by what means is it possible to convey the real charm of his character, his humanity, his culture, and the delicacy of a temperament whose physical reserves of strength were small? Few knew him, few appreciated him: he was not of the kind that gets much recognition from the world. Yet such men as he are the true ornaments of the profession, and on such qualities as theirs half the charm of social life depends.

To Mrs. Maitland, whom he married less than five years ago, we offer very sincerely and respectfully our sympathy. His health gave cause for serious anxiety ever since his last holiday abroad with her, from which he returned just before the outbreak of war. On her has fallen the burden of many anxious months; for there soon became little doubt that pernicious anæmia had supervened. Of the profession's loss in Dr. Maitland we have already spoken; of Mrs. Maitland's we only venture to say that it will be realised by any who came at all under the delightful spell of her husband's personality.

